# Effects of Tinnitus on Cochlear Implant Programming

**DOI:** 10.1177/2331216519836624

**Published:** 2019-03-17

**Authors:** Robert H. Pierzycki, Charlotte Corner, Claire A. Fielden, Pádraig T. Kitterick

**Affiliations:** 1 National Institute for Health Research Nottingham Biomedical Research Centre, UK; 2 Hearing Sciences, Division of Clinical Neuroscience, School of Medicine, University of Nottingham, UK; 3 Nottingham University Hospitals National Health Service Trust, Queen’s Medical Centre, UK

**Keywords:** surveys and questionnaires, appointments and schedules, confusion, attention, audiometry

## Abstract

Clinical observations suggest that tinnitus may interfere with programming cochlear implants (CIs), the process of optimizing the transmission of acoustic information to support speech perception with a CI. Despite tinnitus being highly prevalent among CI users, its effects and impact on CI programming are obscure. This study characterized the nature, time-course, and impact of tinnitus effects encountered by audiologists and patients during programming appointments. Semistructured interviews with six CI audiologists were analyzed thematically to identify tinnitus effects on programming and related coping strategies. Cross-sectional surveys with 67 adult CI patients with tinnitus and 20 CI audiologists in the United Kingdom examined the prevalence and time-course of those effects. Programming parameters established at CI activation appointments of 10 patients with tinnitus were compared with those of 10 patients without tinnitus. On average, 80% of audiologists and 45% of patients reported that tinnitus makes measurements of threshold (T) levels more difficult because patients confuse their tinnitus with CI stimulation. Difficulties appeared most common at CI activation appointments, at which T levels were significantly higher in patients with tinnitus. On average, 26% of patients reported being afraid of “loud” CI stimulation worsening tinnitus, affecting measurements of loudest comfortable (C) stimulation levels, and 34% of audiologists reported observing similar effects. Patients and audiologists reported that tinnitus makes programming appointments more difficult and tiresome for patients. The findings suggest that specific programming strategies may be needed during CI programming with tinnitus, but further research is required to assess the potential impact on outcomes including speech perception.

## Introduction

Patients and clinicians have identified the treatment of tinnitus in people with severe-to-profound deafness as one of priorities for further research (Hall, Mohamad, Firkins, Fenton, & Stockdale, 2013). Tinnitus is commonly defined as the perception of sound in the ear(s) or within the head that occurs in the absence of an external stimulus (Baguley, McFerran, & Hall, 2013; National Institute for Health and Care Excellence, 2017). Cochlear implantation has been proposed as a potential intervention for tinnitus in this population due to the suppressive effects that electrical stimulation can have on tinnitus (Tyler et al., 2008). A cochlear implant (CI) is a hearing prosthesis that conveys auditory information by stimulating spiral ganglion cells within the cochlea with electric pulse trains delivered by a surgically inserted electrode array (Loizou, 1998). Cochlear implantation is already an effective and established intervention for restoring useful aspects of hearing such as the ability to understand speech in people with severe-to-profound hearing loss (UK Cochlear Implant Study Group, 2004). A recent systematic review has suggested that CI use has the potential to alleviate the burden imposed by tinnitus (Ramakers, van Zon, Stegeman, & Grolman, 2015). Epidemiological studies have also found tinnitus-related distress to be lower in CI users than in potential candidates to receive a CI and that the effect on distress is associated with tinnitus being perceived less frequently by CI users (Pierzycki et al., 2016).

Tinnitus occurs in approximately 80% of candidates for implantation (Baguley & Atlas, 2007) and is reported by around 50% of CI recipients (Pierzycki et al., 2016). One potential effect of tinnitus in CI recipients is that its percept may interfere with the process of programming the device. Following implantation surgery, an audiologist alters the parameters of the electrical stimulation to optimize the transmission of acoustic information. These parameters include threshold (T) levels and maximum comfortable (C) levels for each electrode that establish the lower and upper bounds of electrical stimulation, often referred to as the patient’s MAP (Vaerenberg et al., 2014). Clinical observations suggest that measuring T levels behaviorally in CI recipients can be affected by tinnitus (Craddock, 2006).

Tinnitus effects on measuring behavioral thresholds are not unique to CI programming. It has long been known that tinnitus can also cause artifacts when measuring conventional air-conduction hearing thresholds (pure-tone audiometry; Douek & Reid, 1968). As a result, specific recommendations related to tinnitus have been included in audiological practice guidance for conducting pure-tone audiometry (American Speech-Language-Hearing Association, 2005; British Society of Audiology, 2011b; Tunkel et al., 2014). CI programming procedures are not standardized (Vaerenberg et al., 2014), and it is therefore not clear how audiologists deal with tinnitus-related effects during programming appointments, should they arise. It is possible that these effects may impose a burden on the clinicians who program CIs because complete suppression of tinnitus is only likely to be achieved in about half of CI recipients at most (Ramakers et al., 2015).

Tinnitus may also affect the measurement of C levels. CI recipients with tinnitus may try to avoid “loud” stimulation on electrodes where higher current levels could potentially exacerbate tinnitus leading to overconservative limits being placed on the electrical stimulation (Tyler & Baker, 1983). Similar effects have been acknowledged in audiological practice guidance on the assessment of uncomfortable loudness levels (or threshold of discomfort) and real-ear measurements during hearing aid fitting (British Society of Audiology, 2011a; British Society of Audiology and British Academy of Audiology, 2008). In implant recipients, the effects of tinnitus on T or C levels could also be problematic if they lead to a reduction of the range of stimulation delivered across the CI electrodes; that is, the electric dynamic range (EDR). Poorer speech perception in CI recipients have been associated with more variable T levels, lower mean C levels, and smaller mean EDR sizes across the electrode array (Pfingst & Xu, 2005; Pfingst, Xu, & Thompson, 2004). Identifying and characterizing the effects of tinnitus on CI programming could therefore be of substantial clinical importance because improving speech perception remains the primary intended effect of cochlear implantation (National Institute for Health and Care Excellence, 2009; Vaerenberg et al., 2014).

There is lack of systematic research on both the effects that tinnitus has on the process of programming a CI and the strategies that audiologists employ to complete the programming process in adult CI recipients with tinnitus. The first objective of the present study was therefore to explore and characterize those effects and strategies using semistructured interviews with experienced audiologists working across two large CI clinics. The frequency with which audiologists and CI recipients encounter tinnitus-related effects with CI programming is also unknown, as is the time-course over which tinnitus interferes with the process of refining T and C levels to the individual patient. The second objective was therefore to assess the prevalence and impact of those effects using cross-sectional surveys of adult CI recipients and audiologists. The third objective was to investigate the specific hypothesis that CI programming parameters obtained during clinical appointments (T and C levels) differ in patients with tinnitus from those without tinnitus.

## Methods

### Data Collection

The study comprised three parts. In the first part, six audiologists from two major implant centers in the United Kingdom, the Nottingham Auditory Implant Programme (NAIP) and the Midlands Hearing Implant Programme, were interviewed to characterize tinnitus-related effects during CI programming appointments. Purposive sampling was used to recruit senior audiologists who specialized in the postoperative management of adult CI recipients. The interviews lasted for approximately one hour, and audio recordings were made for later transcription and then destroyed. Interviews were conducted in person wherever possible or by telephone where the clinical schedule of the interviewee did not allow for a face-to-face meeting to be held within the time frame of the study.

Two cross-sectional surveys were conducted in the second part of the study to further capture whether audiologists and patients experience tinnitus-related effects that cause difficulties during CI programming appointments, how frequently they occur, and over what time-course. A national survey targeting audiologists with experience of managing adult CI patients across all 18 auditory implant centers that provide services to adults in the United Kingdom was advertised through the British Cochlear Implant Group. CI audiologists in the United Kingdom would typically have experience of programming devices from three major manufacturers: Cochlear Ltd., Advanced Bionics, and Med-El. Twenty audiologists from nine centers, representing 50% of adult CI services in the United Kingdom, took part in the survey. An invitation to participate in a postal survey to explore the potential burden arising from tinnitus after implantation was also sent to all adult CI recipients from the NAIP of whom 128 (20%) responded to the survey. Of the 128 respondents, 67 (52%) patients reported experiencing tinnitus. The proportion of survey respondents with tinnitus was almost identical to and representative of the proportion of CI users experiencing tinnitus (50%) found in a population study in the United Kingdom (Pierzycki et al., 2016). The 67 participants who reported experiencing tinnitus were asked to report whether they experienced tinnitus-related difficulties during routine programming appointments.

In the third part, programming parameter data (e.g., T and C levels) were extracted from the clinical notes of a group of 20 CI users who had given written consent for their clinical records to be accessed. Only data from recipients of Cochlear Ltd. devices were extracted as both T and C levels are measured routinely as part of the recommended programming procedure of those devices, rather than set automatically during programming appointments (cf. Advanced Bionics Corp., 2006), and therefore had the potential to be influenced by tinnitus. The data were extracted from the records of CI activation appointments only as tinnitus effects were reported to be most common at these appointments based on the findings from parts 1 and 2 of the current study. Half of the participants were selected to have ‘never’ experienced tinnitus, while the other half were selected because they had tinnitus that had either started before CI surgery or after CI surgery but before CI activation. Patients who reported that their tinnitus started after CI activation were excluded from the analysis.

### Consent and Ethics Approval

The study followed the principles of the Declaration of Helsinki. Audiologists consenting to participate in the interviews also gave written consent to include their data in the study. Completion of the survey was taken as informed consent to participate. Separate written consent was obtained to access the clinical notes of CI patients participating in the postal survey. The study was approved by the National Research Ethics Service Committee South East Coast—Surrey, United Kingdom.

### Interview Design

**Figure 1.**
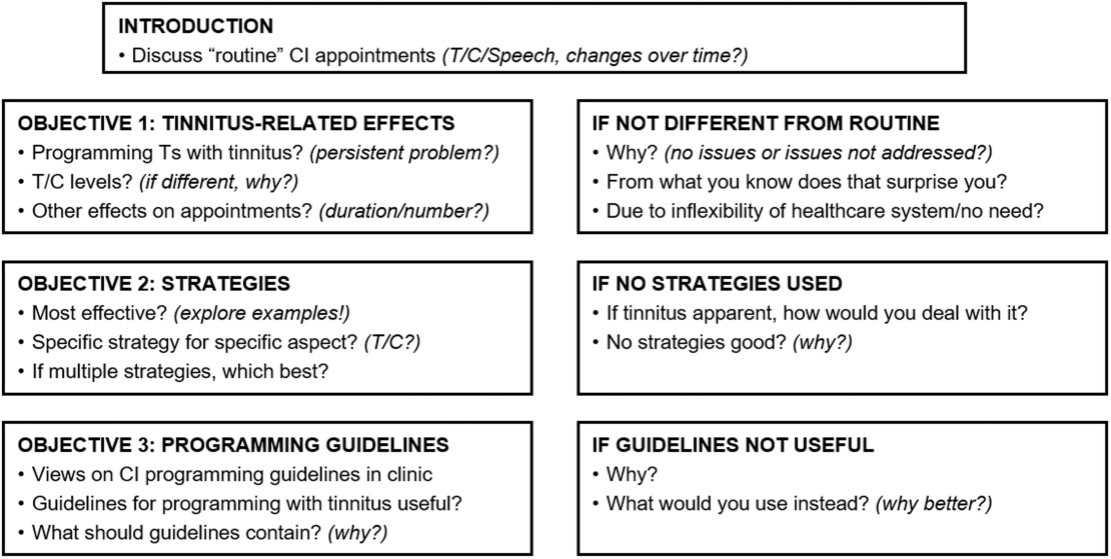
Schematic of the interview schedule. CI = cochlear implant; T = threshold; C = comfortable.

A schematic of the interview schedule is shown in Figure 1. A semistructured approach was adopted to allow the audiologists to identify and explore the issues that they consider to be of most importance while also ensuring that the interview covered specific topics of interest. An initial set of topics was refined by conducting a pilot interview with a CI audiologist involved in the management of adult CI recipients. The final interview topics were (a) the elements of programming appointments that are affected by tinnitus, (b) the strategies that are considered to be most effective in dealing with tinnitus during programming appointments, and (c) the need for guidelines on how to program CIs in adults with tinnitus.

### Survey Design

Information about programming difficulties related to tinnitus was collected from audiologists and CI users as part of surveys exploring the burden from tinnitus after cochlear implantation in the U.K. population (Pierzycki et al., 2016). Survey questions about programming difficulties due to tinnitus were presented to all audiologists and to those CI users who reported experiencing tinnitus. The survey questions are listed in Table 1. The response options were either ‘Yes’/‘No’ or a 5-point Likert scale with choices of ‘Strongly agree’, ‘Agree’, ‘Neither agree nor disagree’, ‘Disagree’, or ‘Strongly disagree’. Responses to questions using the Likert scale were coded as ‘Yes’ if either ‘Strongly agree’ or ‘Agree’ was selected and ‘No’ if either ‘Strongly disagree’ or ‘Disagree’ was selected.

**Table 1.** Survey Questions on Tinnitus-Related Effects During Programming Appointments.

Item		Response mode
Patient survey:		
1.	I was afraid that loud stimulation sounds might make my tinnitus worse.	Likert scale
2.	I found it difficult to tell whether the sounds I heard were coming from the implant or were my tinnitus.	Likert scale
3.	I was tired at the end of my programming appointment because of my tinnitus.	Likert scale
4.	My programming appointment was more difficult because of tinnitus.	Likert scale
Audiologist survey:		
5.	Patients are afraid that loud stimulation sounds might make their tinnitus worse.	Likert scale
6.	Patients find it difficult to tell whether the sounds they hear are coming from the implant or are their tinnitus.	Likert scale
7.	Patients are tired at the end of their programming appointment because of their tinnitus.	Likert scale
8.	Programming appointments are more difficult because of tinnitus.	Likert scale
9.	Have you ever given any specific advice to your cochlear implant patients about their tinnitus during routine appointments? If yes, please describe why and give examples of advice.	Yes/No
10.	Do you sometimes have to use specific procedures or change routine procedures due to tinnitus during routine programming appointments? If yes, please describe why and give examples of changes.	Yes/No

The survey asked respondents to agree or disagree with statements about the potential effects on the measurement of T and C levels due to tinnitus. Respondents were then asked to judge whether these effects, if present, had an impact in terms of creating difficulties with programming and increasing the level of tiredness experienced by the CI patient. Audiologists were asked to report if they experienced these effects and impacts at three points in time: at the first CI activation, at follow-up appointments between 6 and 12 months after first activation, and at follow-up appointments >12 months after first activation. CI recipients were asked to report whether they personally experienced these effects and impacts at first activation, at follow-up appointments between 6 and 12 months after first activation, and at their most recent appointment. The 6- and 12-month cutoffs were chosen as routine time points for appointments following CI activation and because the alleviation of tinnitus and related symptoms has been found to occur on average within 6 to 12 months following implantation (Ramakers et al., 2015).

Two additional questions were included in the audiologist survey to assess their views on the need to provide advice on managing tinnitus to patients and the need to use specific programming procedures for patients with tinnitus. The responses to these questions were compared with the analogous findings from the interview data.

### Analysis

The interviews were coded using the NVivo 10 software (QSR International, Melbourne, Australia) and analyzed thematically following the methodology outlined by Braun and Clarke (2006). The first phase of the analysis method was a verbatim transcription of the interviews. Transcription was done by a single researcher to achieve an in-depth familiarization with the data and to reduce the chances of misinterpretation during the later analysis phases. As no strong hypotheses could be formulated a priori due to the scarcity of studies on the topic, initial ‘codes’ identifying interesting elements of the data set were defined inductively to allow an exploratory data-driven analysis of the content (Braun & Clarke, 2006). Codes were based on keywords or statements that were repeated across the data set. An example of the coding strategy can be seen in the following extract that was initially coded for ‘Distraction,’ ‘Different strategies to overcome tinnitus effects,’ ‘T levels with tinnitus,’ and ‘Accuracy’:

*… it’s almost like a distraction technique really … But there are some people you can do loads of [different techniques to overcome tinnitus effects] and I would say there’s many a time when you can’t be a hundred percent sure that you’ve got an accurate T level.*

In the next phase of the analysis, the codes were organized into themes and reviewed alongside the entire data set by the same researcher using the following criteria: (a) the collated interview extracts are representative of and support the themes, (b) the themes are consistent with the overall narrative when compared against the entire data set, and (c) the assessment of potential relationships between the themes confirms a theme’s independence or suggests the emergence of a dominant, overarching theme or a set of related subthemes. The themes and subthemes were then reviewed by two other researchers, one of whom had also undertaken an independent familiarization with the data set by an in-depth reading of the interview transcripts. The final choice and naming of themes was arrived at by consensus among the three researchers.

Descriptive statistics were used to summarize the prevalence of tinnitus-related effects and impacts on programming appointments reported in the surveys, separately for audiologists and CI users. The comments from audiologists on the reported tinnitus-related advice and specific programming strategies used to overcome programming difficulties due to tinnitus were compared with those found in the interview data.

For the analysis of programming data, T and C levels for each active electrode of each patient were extracted from their earliest programming MAP. Their earliest MAP was defined as that which was measured at their first CI activation appointment and subsequently programmed into their speech processor. The T and C levels reported by the programming software Custom Sound (Cochlear Ltd., Sydney, Australia) were first converted into microamperes using conversion formulae obtained from the manufacturer and then to decibels (dB re 1 mA; Pfingst & Xu, 2005). The size of the EDR in dB was then derived for each active electrode by subtracting its T and C levels; that is, EDR = C – T. The resulting T, C, and EDR values of patients with and without tinnitus were compared using generalized estimating equations (GEE) in SPSS v.24 (IBM Corp., Released 2016). The GEE approach accounted for the fact that T, C, and EDR values were likely to be similar (i.e., not independent measurements) across a given patient’s electrode array due in part to the use of interpolation. Three separate GEE models were run to test the hypothesis that Ts, Cs, and EDRs differ between the groups of patients with and without tinnitus. The effects on T and C levels were analyzed, in addition to those on the EDR, to test the specific reports of effects on those parameters made by audiologists in the interviews and by patients and clinicians who responded to the surveys. Results were considered statistically significant if *p* < .05.

## Results

### Audiologist Interviews

**Figure 2.**
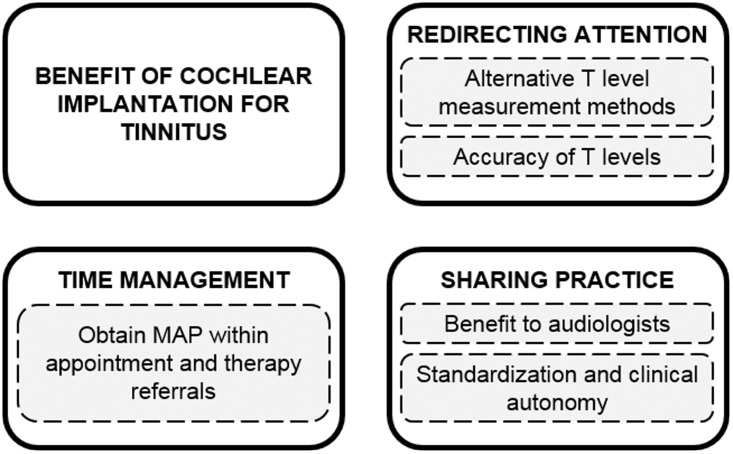
Identified themes (solid) and subthemes (dashed) from the qualitative analysis of the audiologist interviews. T = threshold.

Figure 2 shows the four themes identified through the thematic analysis. The themes and subthemes are described in the following sections with supporting extracts (in italics) from the interviews of the six audiologists (A1–A6).

#### Theme 1: Benefit of cochlear implantation for tinnitus

A common backdrop narrative throughout all interviews related to the recognition by the audiologists that they commonly observe the alleviation of tinnitus following cochlear implantation. This benefit to tinnitus was expressed in different ways, either as a degree of suppression of the tinnitus sound itself (A6: *most patients do get some suppression with the implant*)**or more commonly as a reduced awareness of tinnitus (A1:* It’s not like [tinnitus] disappears altogether, it’s almost more that [patients] can cope better with it or they don’t notice it as much*).**The reduced awareness was also associated with the presence of electric stimulation (A2:* very often once you begin electrical stimulation [patients’] awareness of the tinnitus goes away*; A3: *a lot of [patients] will say when [the implant] is off that they’re aware of their tinnitus*).**However, audiologists also reported that tinnitus can be bothersome despite using a CI (A3:* There are … individuals … who despite having had an implant still complain that they are really bothered by tinnitus*)**and that tinnitus can worsen in some CI recipients after implantation (A1:* we’ve had people who’ve had their tinnitus made definitely worse from having the operation [to surgically insert the implant]*).

#### Theme 2: Redirecting attention

All audiologists reported experiencing difficulties due to tinnitus during CI programming. The audiologists focused primarily on the disruptive effects that a patient’s awareness of their tinnitus can have on T level measurements. They reported that their patients become aware of their tinnitus during appointments when the implant is switched off and they are encouraged to focus on soft sounds during measurements of T levels:

*once [patients] have their implant [switched] off, you get a large number of people saying ‘oh now I can hear my tinnitus again’ … So even though they weren’t complaining about [tinnitus] when they first came in, not having the stimulation [from the CI on] and … asking them to focus on really tiny quiet sounds seems to bring back their tinnitus, at least for the duration of the testing.* (A1)

or as expressed by audiologist A3:

*you’re trying to get [patients] to focus on a sound … , you’re getting them to sit in silence … Most of the day they’re having their implant [switched] on. You … hook them up to the computer, they’ve got no other sound, they’re profoundly deaf, it’s completely silent and you’re asking them to focus on [programming stimuli] bleeps  … it doesn’t help tinnitus that sort of situation where they’re putting all their focus on listening to little [soft] sounds …  it seems to make it worse [for tinnitus].*

However, some audiologists also reported specific issues with programming in CI recipients who perceive their tinnitus during programming appointments even though their CI is switched on (A1: *in a case of severe tinnitus … Programming is very, very difficult … because the tinnitus is there all the time whether the implant’s on [or], … off*). The audiologists also described situations in which restored awareness of tinnitus during T level measurements can lead to confusion between tinnitus and the stimuli being presented:

*[patients are] tapping away [with a pen to signal hearing the stimulus] and I’m not doing anything [with stimulus presentation] … either their tinnitus will mirror the sound that you’re playing or tinnitus [sounds the] same … so [tinnitus] just keeps going and going, and going so it can be more difficult.* (A4)

Distinguishing between the stimulus and the patient’s tinnitus also appeared to be more difficult when the pitch of the presented stimulus resembles the perceived pitch of tinnitus (A5: *you’ll often find … certain electrodes which match up nicely to the tinnitus pitch and [patients] find those ones difficult*). However, one audiologist was of the opinion that difficulties are not specific to a particular pitch or electrode (A3: *[tinnitus-related difficulty] doesn’t just occur in the higher pitch MAPs because perhaps that’s where the tinnitus is, it seems to be all over [different pitches or electrodes]*).

Evidence of tinnitus-related effects on the measurement of C levels was limited. When asked about C level measurements directly, the audiologists did not feel that this aspect of programming was particularly affected by tinnitus (A3: *I’m not sure that [tinnitus] does necessarily [affect C levels]*;* A2: I wouldn’t say [measuring C levels] is particularly different when they have tinnitus*; *A6: I’ve never known tinnitus to have an effect on comfort levels*). Two audiologists did acknowledge that an effect on C levels may be possible, but this was not supported by examples of particular experiences during appointments (A4: *theoretically you could get the patients saying ‘well if you make [the stimulus] loud you’re going to aggravate my tinnitus’ so they don’t want to make [stimulation] loud but I can’t think of anybody who’s actually said that to me*; A5: *The only time I would say that you’ve got issues is that [tinnitus] has just generally … made [patients] more hypersensitive and anxious about things … when you start pushing the limits of what they can tolerate loudness wise*).

#### Subtheme: Alternative T level measurement methods

Audiologists described various ways of turning the patient’s attention away from tinnitus as effective strategies for dealing with tinnitus-related difficulties during T level measurements. For example, one audiologist commented that alternating between different assessments is helpful (A1: *tinnitus is less of a problem if you switch from T to C levels*) and that they would also alternate between threshold and suprathreshold stimulation to overcome tinnitus-related difficulties (A1: *I would move away from the threshold levels and onto the louder levels … and then go back to the threshold levels to see if you’ve … moved their attention away [from tinnitus] slightly*). Other audiologists reported repeating stimulus bursts and asking patients to not only report when but also how many sounds they hear, for example (A4: *[strategies include] changing the number of beeps or getting [patients] to count one, two, three beeps*). Some audiologists reported that asking patients to count stimulus bursts could also be used to ascertain the accuracy of measurements (A2: *if you want to be sure about a T level … then yes you would ask to count the beeps so you know it’s a true threshold … that’s standard practice*). Three audiologists reported alternating stimulation between the low- and high-frequency electrodes as an effective strategy to disrupt the patient’s focus on their typically high-pitched tinnitus:

*if tinnitus, say, is particularly high-pitched and [patients] are having a lot of trouble with the high-pitched T levels then I might change … the frequency to a more apical electrode where it’s a deeper sound … to try and get their attention away from that continual sound they’re hearing.* (A1)

or audiologists would move across different parts of the electrode array:

*I would certainly move to a different part of the electrode array … start in the middle and instead of then laboriously go through [the electrode array], move around and then [patients] are getting a novel stimulation from … a different pitch*. (A2)

However, some audiologists were of the opinion that changing the electrode channels would not make a difference.

*[The tinnitus effect] seems to be consistent across all [electrodes] so I’m not sure whether jumping around would make any difference … you sometimes do that anyway as part of the mapping but it doesn’t seem to have a big influence*. (A3)

#### Subtheme: Accuracy of T levels

The audiologists admitted that the difficulties they experience with measuring thresholds in the presence of distracting tinnitus are common in audiology and viewed them as “normal.” Although they reported that strategies to redirect attention can help overcome these difficulties, they still voiced concerns over the reliability and accuracy of the resulting threshold measurements. The potential effect of tinnitus on the reliability of thresholds was recognized as a known issue in pure-tone audiometry (A3: *generally [tinnitus] interferes when … doing … threshold measures, … like [tinnitus] interferes with audiometry*; A2: *tinnitus is interfering when you’re measuring T levels … this is the same with [non-implant] audiology when you’re trying to measure a threshold*). Some audiologists acknowledged potential issues with the accuracy of measured T levels (A1: *there’s many a time when you can’t be a hundred percent sure that you’ve got an accurate T level*; A6: *if [patients] have got bad tinnitus it makes setting thresholds … more unreliable or less reliable because you probably have to go supra-threshold to get the … threshold above the level of the tinnitus*), but this effect of tinnitus was not always regarded as a major issue during CI programming (A2: *I’m not ruling [tinnitus] out it’s just [tinnitus] is not that often that it would become an issue*).

#### Theme 3: Time management

When asked about other effects of tinnitus on programming appointments, the audiologists reported that tinnitus-related difficulties have the potential to increase the length of time required to program a CI, for example, due to the false responses associated with a patient confusing their tinnitus percept with stimulus presentations (A1: *you’re clearly getting a lot of false positive responses on your threshold measurements [and] the session definitely takes longer*). They identified effective time management as a key skill that is necessary to obtain MAPs within the allocated appointment time and to avoid the need to make repeated measurements.

#### Subtheme: Obtain MAP within appointment and therapy referrals

The discussion around effective time management strategies was summarized in a subtheme on the necessity to complete programming of MAPs within the appointment. Repeating measurements or extending appointments were regarded as counterproductive strategies (A4: *if they can’t do Ts … there’s no point in making them not do Ts very well for half an hour or whatever, you just get what you can get*) and potentially tiring the patients and aggravating their tinnitus (A4: *the longer you keep going at it the more tired the patient gets or the worse the tinnitus plays up*).

Despite pressures on their time, audiologists seemed to be confident in their time management strategies (A1: *I would find ways within my hour to deal with the tinnitus*) but emphasized the importance of prioritizing to address other patient’s issues during the appointment (A5: *with x amount of time you prioritize what’s important and work with that. And that’s why your discussion with the patient at the start is really important because … you need to address any sort of issues they’ve got*).

Audiologists also considered the interpolation of intermediate T levels between those actually measured as a useful strategy to complete the programming process within the available time (A1: *people [with tinnitus] can take longer to program … if I can see that they’re having a lot of trouble then I will go to interpolated levels … it’s probably the best you can do given the inconsistency in responses*). The audiologists agreed that CI recipients with tinnitus would gain more from an extended hearing therapy than from extended or additional programming appointments (A1: *if [tinnitus] is a consistent problem it won’t be any better the next time*; A6: *we’ve got … a good hearing therapy service here so if there was an issue with tinnitus I don’t think the solution would lie in [extended] programming, [but] in getting [the patients] to see the hearing therapists*).

#### Theme 4: Sharing practice

The fourth theme reflected the views of the audiologists around the need for practice guidance on CI programming in recipients with tinnitus. The audiologists generally saw a greater value in having access to a “resource” that would allow the sharing of best practice on strategies for overcoming tinnitus-related difficulties during programming appointments rather than in a prescriptive set of guidelines.

#### Subtheme: Benefit to audiologists

Four audiologists agreed that guidance on programming of CI recipients with tinnitus would be of some benefit. Guidance was seen as a potentially useful method of sharing approaches to patient management among both new and experienced audiologists (A1: *Yes, I do think [shared guidance] would be useful because it might give you a few more things to try … Particularly if … people … are new in the field*). The availability of a written guide was seen as particularly useful for less experienced audiologists (A2: *it would be helpful if there was more … written down … for [new audiologists] … so they’re not completely baffled by [tinnitus-related difficulties]*). The role of guidance was viewed consistently as an “ideas bank” that could support clinical practice or judgment (A1: *[guidance] could give us some good suggestions on how to alter your programming technique … even little things … about changing the number … or … frequency of the beeps, … so you could have more confidence in your results*).

#### Subtheme: Standardization and clinical autonomy

The potential benefits of guidance were contrasted with a need to retain a flexibility in how the audiologists choose to manage individual patients. Although guidelines have the potential to standardize clinical practice (A4: *I suppose it’s a way of standardizing*), the audiologists felt strongly that any programming guidelines should not restrict clinical autonomy required to address the individual patient’s needs:

*I think if you contrast [guidelines] with something like [British Society of Audiology] recommended procedures for … [non-implant] audiometry, I would expect people to adhere to those because that’s … the agreed standard. Cochlear implants are slightly different and there’s no recommended procedure … so people would find it useful to have guidelines, but I think they’d still find it useful to be able to use their own experience. Every patient’s different … so you’ve got to be very adaptable*. (A2)

In the opinion of one audiologist, patient heterogeneity would make the task of creating guidance infeasible (A6: *[the potential issues are] so heterogeneous … I really can’t see how you would go about writing a guideline for it*). The task of providing useful advice that could be generalized to all patients with tinnitus was also thought to be hindered by differing programming recommendations and programming strategies across CI makes and models (A2: *because the way we program different implants is slightly different [with different CI manufacturers]* … *they might have different opinions about what to do about tinnitus).* The importance of the audiologists’ contribution and experience to the programming process was emphasized as crucial in formulating any form of guidance (A2: *the manufacturers need to be included in the consultation but it’s got to come from experienced clinicians)*.

### Audiologist and Patient Survey

#### Tinnitus-related difficulties

The demographics of CI users with tinnitus participating in the patient survey are listed in Table 2. The majority (70%) of patients were implanted with devices from Cochlear Ltd., while 27% and 3% of patients used devices from Advanced Bionics and Med-El, respectively. The self-reported duration of CI use was 7.5 years on average (standard deviation, SD = 6.8) with an average duration of deafness before implantation of about 13 years (*SD* = 14.7). About 84% of patients reported that the onset of their tinnitus occurred before CI surgery, while 5% of patients reported that tinnitus started after implantation but before implant activation, and about 11% of patients reported to have developed tinnitus after CI activation.

**Table 2.** Demographics of CI Users With Tinnitus Participating in the Patient Survey (Missing Data Excluded in %).

Characteristic	Demographic	
	*N*	%
Total	67	100
Males	26	39
Unilateral CI users	65	97
CI make		
Cochlear Ltd.	46	70
Advanced Bionics	18	27
Med-El	2	3
Missing	1	–
Tinnitus onset		
Before CI surgery	54	84
After CI surgery, before activation	3	5
After CI surgery, after activation	7	11
Missing	3	–
	*Mean*	*SD*
Age (years)	53.9	19.0
Duration of deafness (years)	13.0	14.7
CI use experience (years)	7.5	6.8

*Note.* CI = cochlear implant; SD = standard deviation.

**Figure 3.**
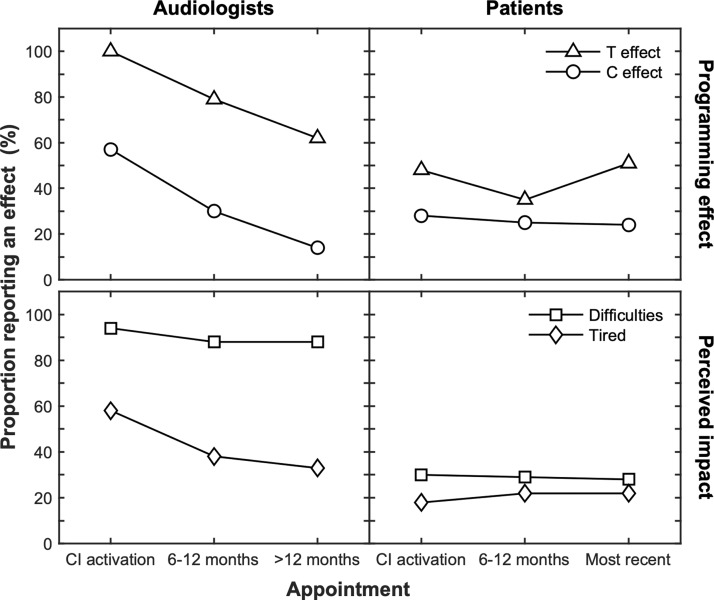
Tinnitus-related effects during programming appointments reported in the cross-sectional survey of audiologists (left) and patients (right). Panels in the top row show the effects on measuring T (triangles) and C levels (circles). Panels in the bottom row show the reported difficulties with programming (squares) and tiredness (diamonds) due to tinnitus. CI = cochlear implant; T = threshold; C = comfortable.

Figure 3 shows the proportions of audiologists and patients reporting tinnitus-related effects and impacts on programming appointments. Both audiologists and patients agreed that the tinnitus percept can be confused with the stimuli presented during CI programming when estimating T levels. This effect was observed by all audiologists at the CI activation appointment, but fewer (62%) reported observing it >12 months after first activation. On average, 45% of CI users with tinnitus reported confusing stimuli used to measure T levels with their tinnitus, regardless of when the appointment took place. Both groups also agreed that being afraid of loud stimulation can be associated with patients being afraid of worsening tinnitus. Similar to the interview findings, this effect on C levels was reported by the audiologists to be less common (57% at activation) than the effects on T levels and to diminish with time (about 14% at appointments >12 months following activation). An effect of tinnitus on C level measurements was reported by 24% to 28% of CI users with tinnitus, remaining almost unchanged regardless of their duration of CI use.

The reported levels of perceived difficulty with programming due to tinnitus differed substantially between audiologists and CI users. Nearly all audiologists (88% to 94%) agreed that tinnitus makes programming more difficult at all appointments. However, only 28% to 30% of patients with tinnitus agreed that tinnitus makes CI programming more difficult. When asked whether tinnitus increases the level of tiredness in patients, about 33% of audiologists and 22% of CI users agreed that even experienced implant users (>12 post CI activation) can feel more tired at the end of their programming appointments due to tinnitus.

#### Managing tinnitus and programming difficulties by audiologists

The majority of audiologists (89%) reported providing specific advice about tinnitus to their CI patients during routine appointments. The advice included tips on how to reduce the impact of tinnitus and various tinnitus management strategies, for example, sound enrichment therapy, stress management, and relaxation. About 29% of those audiologists also considered referring their patients for further advice or therapy for tinnitus such as counseling or a visit to a specialist tinnitus clinic.

Almost all (95%) of audiologists also reported using specific CI programming strategies to overcome tinnitus-related difficulties during programming appointments. The reported strategies are summarized and compared against those reported in the interview data in Table 3. The most commonly used strategy was varying the number of presentation stimuli, either with or without a change in the task from reporting sound detection to counting the number of stimulus presentations. The common narrative was that the majority of strategies were specifically used to support the measurement of T levels and were largely consistent with those strategies reported in the interviews. The survey data also identified additional strategies not mentioned in the interview data, such as the potential use of stimulation with speech in ‘live’ mode for measuring T levels, the use of objective measures, and introducing additional breaks to reduce the burden on the patient.

**Table 3.** Strategies Used by Audiologists to Overcome Programming Difficulties due to Tinnitus During Programming Appointments Reported in the Interviews and Cross-Sectional Survey.

Audiologist strategy		Survey (*n*)	Interviews
1.	Vary/count the number of stimulus presentations	7	Yes
2.	Loudness scaling/balancing	3	Yes
3.	Present suprathreshold stimuli or ascending loudness judgments from audible, manually set T levels, or alternate between T and C measurements	3	Yes
4.	Alternate between stimulation electrodes/sites; for example, basal then apical, then basal again, and so on	2	Yes
5.	If tinnitus particularly bad and measurement not possible: reduce time spent on T measurements or no changes to T levels, but checking C levels	2	Yes
6.	Set/verify Ts based on free field aided responses rather than psychophysics (potentially using narrowband noise instead of warble tones for aided testing)	2	Yes
7.	Set Ts based on live/speech measures or adjust based on detection levels with mics on	2	No
8.	Measuring on only selected (widely spaced) electrodes and interpolation	1	Yes
9.	Use objective measures	1	No
10.	Offer breaks	1	No

### Comparison of Programming Parameters

**Figure 4.**
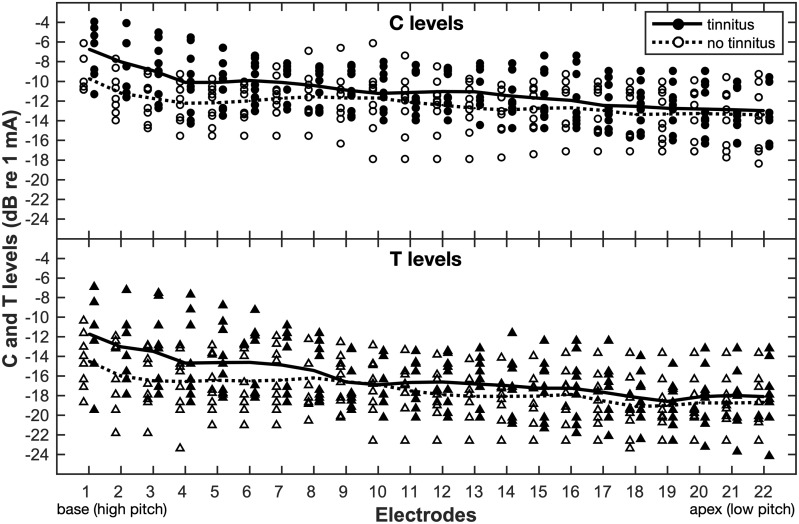
The T and C levels derived for the active CI electrodes during first CI activation appointments in patients with (filled) and without tinnitus (open). The lines represent the average across the levels obtained for each electrode in the tinnitus (solid) and no tinnitus group (dashed). All patients were recipients of Cochlear Ltd. devices. T = threshold; C = comfortable.

Table 4 lists demographic information for the 20 CI users included in the analysis of programming parameters measured during CI activation appointments (more detailed demographics can be found in the supplemental Table S1). The tinnitus and nontinnitus groups were similar with respect to age at implantation and self-reported duration of deafness (Mann–Whitney U test, *p* > .05). Figure 4 shows the extracted T and C levels across all active electrodes measured for the 10 patients reporting tinnitus and those who reported never experiencing tinnitus.

**Table 4.** Demographics of CI Users Included in the CI Programming Data Analysis (Missing Data Excluded in %).

Group	All		No tinnitus		Tinnitus	
	*N*	%	*N*	%	*N*	%
Total	20	100	10	50	10	50
Males	10	50	5	50	5	50
Unilateral CI users	20	100	10	100	10	100
Tinnitus onset						
Before CI surgery	–	–	–	–	9	90
After CI surgery, before CI activation	–	–	–	–	1	10
	*Mean*	*SD*	*Mean*	*SD*	*Mean*	*SD*
Age at implantation (years)	57.6	13.7	60.4	13.2	54.8	14.4
Duration of deafness (years)	16.6	14.4	19.8	18.1	13.8	10.2

*Note.* All participants were implanted with devices from Cochlear Ltd. CI = cochlear implant; SD = standard deviation.

The GEE model results showed that T levels were significantly higher on average by 1.7 dB in patients with tinnitus than in those without (*p* = .048). This finding was consistent with the tinnitus-related difficulties during T level measurements reported by audiologists and adult CI patients in parts 1 and 2 of the current study. The mean C level was also found to be higher by about 1.6 dB in patients with tinnitus than in patients without tinnitus, but this difference was not statistically significant (*p* > .51). The size of the EDRs in the two groups was also not significantly different (*p* > .74).

## Discussion

This study used interviews and cross-sectional surveys to explore the experiences of the impact of tinnitus on the process of programming CIs among adult CI recipients and audiologists. The study aimed to characterize the nature, prevalence, and time-course of the tinnitus-related effects that they encounter during programming appointments, to assess the potential impacts of those effects, that is, whether they cause specific difficulties during programming appointments, and to identify the strategies audiologists employ to overcome those effects.

The interviews, surveys, and programming data suggested that tinnitus mostly affects measurements of T levels during CI programming. Patients tend to confuse the test stimuli with their tinnitus, which in turn raises concerns about the accuracy of T levels in this patient group. This finding is consistent with clinical observations that T levels can be set too high when CI recipients are unsure whether they hear their tinnitus or the stimulus through their implant (Craddock, 2006). The reported effects on measuring T levels in CI users were similar to the potential effects of tinnitus on threshold measurements obtained using pure-tone audiometry anticipated in published clinical guidance (British Society of Audiology, 2011b). Compatible with these expectations and findings, T levels obtained from the clinical notes of patients reporting tinnitus were observed to be significantly higher than those obtained in patients without tinnitus. Thus, the tinnitus-related programming effects reported by both CI users and audiologists appear to have a measurable effect on the parameters that are used to determine the pattern of stimulation delivered through a patient’s CI.

A more mixed picture was found for the effect of tinnitus on measuring C levels. Audiologists acknowledged that patients might be anxious about “loud” stimulation but reported that any such effects quickly diminish over time following initial activation. In general, effects on measuring C levels appeared to be far less prevalent than effects on T levels and the comparison of C levels measured at activation appointments between patients with and without tinnitus also did not show a significant effect of tinnitus. The less frequent reporting of effects on C level measurements could be due to patients’ confidence and willingness to try higher levels of stimulation as their tinnitus is being suppressed during suprathreshold stimulation. An alternative explanation is that patients avoid higher stimulation levels during C level measurements because of their tinnitus but do not inform their audiologist that it is tinnitus-related anxiety rather than loudness-related discomfort that is determining their maximum comfort level. The fact that CI stimuli are always audible during C level measurements may also make these effects less noticeable to audiologists than the effects on T levels, where audiologists could plausibly infer the presence of an effect of tinnitus from the false detection of sounds in the absence of stimulation.

The present findings may have potential implications for the management of CI recipients with tinnitus in CI clinics. Audiologists were generally of the view that simply repeating T level measurements during appointments is counterproductive because this may not only fail to improve the final measurements but may also tire the patient and further aggravate their tinnitus. They were also reluctant to recommend additional programming appointments for patients experiencing tinnitus-related difficulties by reasoning that tinnitus is also likely to continue to create difficulties at follow-up appointments. This observation made in the interviews suggested that tinnitus-related difficulties during programming and tiredness persist over time, a possibility that was confirmed by the survey results from both audiologists and patients. This stable effect of tinnitus on programming is consistent with the fact that tinnitus is suppressed mostly during CI stimulation but returns when the stimulation is switched off (Vlastarakos, Nazos, Tavoulari, & Nikolopoulos, 2014; Zeng et al., 2011).

The persistence of tinnitus-related difficulties in programming over time would also explain in part why the audiologists suggested referring patients for additional hearing therapy as more appropriate management option than extending or offering additional appointments to repeat programming measurements. Educating patients about tinnitus during therapy sessions may help them to overcome their negative preconceptions and reduce anxiety about tinnitus (Seidman, 2017). As a result, this therapy could help patients try louder stimulation sounds during C level measurements. However, difficulties with measuring T levels appear to persist even at appointments more than a year after first activation of the CI. This observation is compatible with the suggestion that tinnitus may not be fully suppressed after cochlear implantation (Ramakers et al., 2015) and therefore that additional hearing therapy may not be effective in managing this particular type of programming difficulty.

Given that the interviews, surveys, and programming data consistently suggested that T rather than C levels are most likely to be affected by tinnitus, a time-efficient approach to avoiding tinnitus-related effects on T level measurements would simply be to automatically estimate T levels from C levels. This approach is in fact already the recommended procedure for programming certain makes of CI systems (Craddock, 2006; Wolfe & Schafer, 2014). However, the effect of that approach would be to fix EDRs across electrodes and possibly increase the variability of T levels across the electrode array, which has been shown to be negatively correlated with speech perception outcomes with CIs (Pfingst & Xu, 2005; Pfingst et al., 2004). Therefore, speech outcomes may be maximized by adopting the strategies suggested by the audiologists that seek to maximize the fidelity of any T levels measurements that are obtained within the allocated appointment time.

The specific strategies described by audiologists as effective in dealing with any tinnitus-related difficulties when obtaining MAPs were aimed either at redirecting the patient’s attention away from tinnitus (e.g., counting stimuli) or at reducing tinnitus-related effects by frequent task switching, for example, by alternating between near- and suprathreshold levels, T and C level measurements, or different electrodes. The audiologists also endeavor to avoid spurious measurements and obtain the required programming parameters as quickly as possible, for example, by using interpolation for electrodes eliciting pitch percepts similar to the patient’s tinnitus pitch. These strategies somewhat resemble more general troubleshooting methods used in CI programming practice (Craddock, 2006; Wolfe & Schafer, 2014). Despite the fact that the CI audiologists considered tinnitus interference on threshold measurements as “normal” in audiology, the strategies they employ appear to be far more complex than those mentioned in the available guidance on addressing tinnitus-related effects when conducting pure-tone audiometry (American Speech-Language-Hearing Association, 2005; British Society of Audiology, 2011b).

It is not clear whether the strategies for obtaining reliable T levels would be equally effective for different tinnitus percepts. For example, the method of interpolation or switching electrodes may not overcome or alleviate interference from more spectrally complex or multiple tinnitus percepts (Baguley et al., 2013). If tinnitus affects measurements on multiple or all electrodes, T levels measured on selected electrodes before interpolation could also be affected and overestimated. This possibility reflects the reported experience of the audiologists that tinnitus-related effects may not be specific to only one pitch or electrode, and why the current analysis of programming data appears to show elevated T levels across a range of electrodes. On the other hand, using repetitive stimulus bursts and asking patients to count them may be problematic with tinnitus that has more temporally complex properties, for example, intermittent or pulsatile (Baguley et al., 2013). Therefore, having access to information about the characteristics of the patient’s tinnitus could be useful for determining the strategies likely to help overcome related difficulties during programming appointments.

The use of distraction techniques that involve changing the patient’s task from reporting of “when” a stimulus is heard to “how many” repeated stimulus bursts are heard may have a detrimental effect on the reliability and accuracy of measured T levels in CI patients with attention problems (Castellanos, Kronenberger, & Pisoni, 2018). Irregular changes of stimulus pitch or electrodes may also not be appropriate in these patients due to the increased central processing demands imposed by deviating stimuli (Horvath, Roeber, Bendixen, & Schroger, 2008). Asking the patient to count repeated stimulus bursts may also require higher stimulus levels for detection and therefore lead to higher T levels irrespective of whether tinnitus is present or not. While requiring patients to detect all presented stimulus bursts rather than just one would reduce the number of false responses when stimulation is not present, it also imposes a stricter response criterion that would correspond to a higher point on the psychometric function relating the proportion of correct responses to presentation level (Levitt, 1971). Overall, the present findings suggest that further studies may be needed to test the effectiveness of the reported strategies in overcoming tinnitus-related effects on programming and whether their effectiveness varies with respect to the individual characteristics of the patient and their tinnitus.

Studies in other areas of medicine such as fear and pain management (Ferrell, Ferrell, Ahn, & Tran, 1994; Peretz & Gluck, 1999) have also suggested that distraction techniques may be ineffective in the management of patients with catastrophic beliefs and hypervigilance to the symptoms they experience (Johnson, 2005). They propose that additional therapy may be required to modify these characteristics for the patient to be able to disengage their attention from their symptoms. Catastrophic beliefs, selective attention, and monitoring of tinnitus have been widely recognized as a psychological aspect of tinnitus-related distress (McKenna, Handscomb, Hoare, & Hall, 2014). The additional therapy suggested by the audiologists could aim to reduce the negative thinking, anxiety, and thus hypervigilance to tinnitus during T level measurements to some extent, but there is currently limited evidence that tinnitus interventions can improve the patient’s ability to shift attention from their tinnitus (McKenna et al., 2014). If such effects are desired, the therapy may require a highly structured approach that includes aspects of cognitive behavioral therapy that has been shown to be effective for managing the psychological aspects of tinnitus (Martinez-Devesa, Perera, Theodoulou, & Waddell, 2010). It is not yet clear whether an additional extended tinnitus therapy would be acceptable to CI patients who may already experience some alleviation of tinnitus or who may already undergo a hearing therapy to manage the negative consequences of their profound hearing loss.

Further research should assess the reproducibility of T levels to confirm or reject audiologists’ and patients’ perceptions that tinnitus interferes with the measurement of T levels at different appointments over time and to replicate the current finding that T levels are significantly higher in those with tinnitus. A longitudinal study of tinnitus-related effects may be particularly insightful for clinical practice as the programming process to establish the programming MAPs and EDRs may extend in CI users over about 12-month period after CI activation (Hughes et al., 2001). That research could also provide an opportunity for testing the effectiveness of different programming strategies in overcoming tinnitus-related programming difficulties. While having a “bank of strategies” may be useful to audiologists, such a resource should be supported by evidence-based guidance on how to use those strategies effectively. A key outstanding question remains whether the effects of tinnitus on CI programming, as identified here both qualitatively and quantitatively, ultimately has a negative impact on the speech perception abilities of these CI recipients that is not only measurable but also large enough to be clinically meaningful. The need to conduct research to fully understand the nature and consequences of the effects of tinnitus on CI programming may become even more pressing with the increasing focus on providing CIs specifically for the alleviation of tinnitus.

## Supplemental Material

Supplemental material for Effects of Tinnitus on Cochlear Implant Programming Click here for additional data file. Supplemental Material for Effects of Tinnitus on Cochlear Implant Programming by Robert H. Pierzycki, Charlotte Corner, Claire A. Fielden and Pádraig T. Kitterick in Trends in Hearing

## Supplemental Material

Supplementary Table S1 containing detailed information about CI patients included in the CI programming data analysis is available online.
