# Supplementary material for: Effects of Tinnitus on Cochlear Implant Programming
Source: Trends Hear. 2019 Mar 17;23:2331216519836624. doi: 10.1177/2331216519836624 (PMC6423681; doi:10.1177/2331216519836624)
Supplement: Supplemental material for Effects of Tinnitus on Cochlear Implant Programming [file Supplemental_Material.pdf]

# Effects of tinnitus on cochlear implant programming

**Supplemental Table S1**

Robert H. Pierzycki[1, 2], Charlotte Corner[1, 2], Claire A. Fielden[1, 3] and Pádraig T. Kitterick[1, 2, 3]

[1] *NIHR Nottingham Biomedical Research Centre, Ropewalk House, 113 The Ropewalk, Nottingham NG1 5DU, UK*

[2] *Hearing Sciences, Division of Clinical Neuroscience, School of Medicine, University of Nottingham, Nottingham NG7 2UH, UK*

[3] *Nottingham University Hospitals NHS Trust, Queen's Medical Centre, Nottingham NG7 2UH, UK*

**Correspondence:** Robert H. Pierzycki, NIHR Nottingham Biomedical Research Centre, Ropewalk House, 113 The Ropewalk, Nottingham NG1 5DU, UK. E-mail: robert.pierzycki@nottingham.ac.uk, Phone: +44(0)1158232829, Fax: +44(0)1158232618

**Short title:** Cochlear implant programming with tinnitus

Table S1. Detailed information about CI patients included in the CI programming data analysis. All participants were implanted with devices from Cochlear Ltd., and using ACE coding strategy in MP1+2 stimulation mode.

| ID | Sex | Age at CI (years) | CI ear | N active electrodes | CI type | Sound processor | Duration of deafness (years) | Etiology of deafness | Tinnitus onset |
|---|---|---|---|---|---|---|---|---|---|
| P01 | M | 78 | R | 22 | CI24RE (CA) | CP900 Series | 2 | Noise exposure | No tinnitus |
| P02 | F | 74 | L | 22 | CI512 | CP900 Series | 7 | No data | No tinnitus |
| P03 | F | 74 | R | 22 | CI24RE (CA) | CP900 Series | 6 | Hereditary | No tinnitus |
| P04 | M | 40 | R | 22 | CI24RE (CA) | Freedom | 40 | Unknown | No tinnitus |
| P05 | M | 54 | R | 22 | CI512 | CP900 Series | 25 | Unknown | No tinnitus |
| P06 | F | 47 | R | 22 | CI24RE (CA) | CP810 | No data | Congenital | No tinnitus |
| P07 | F | 65 | R | 22 | CI512 | CP810 | 10 | No data | No tinnitus |
| P08 | M | 58 | R | 21 | CI24RE (CA) | CP900 Series | 2 | Unknown | No tinnitus |
| P09 | F | 47 | R | 19 | CI24RE (CA) | Freedom | 46 | Congenital | No tinnitus |
| P10 | M | 67 | R | 22 | CI512 | CP900 Series | 40 | Ossification | No tinnitus |
| P11 | F | 66 | L | 22 | CI512 | CP810 | 1 | No data | Before CI surgery |
| P12 | M | 77 | L | 22 | CI24RE (CA) | CP900 Series | 7 | Progressive | Before CI surgery |
| P13 | M | 38 | R | 19 | CI24RE (CA) | CP900 Series | 25 | No data | Before CI surgery |
| P14 | F | 44 | R | 20 | CI24R (CA) | ESPrit 3G | 10 | No data | Before CI surgery |
| P15 | M | 60 | L | 22 | CI24RE (CA) | CP900 Series | 8 | Ossification/Infection | Before CI surgery |
| P16 | M | 32 | R | 22 | CI24RE (CA) | CP900 Series | 1 | Cogan syndrome | Before CI surgery |
| P17 | M | 50 | R | 22 | CI24RE (CA) | CP810 | 15 | Otosclerosis/Noise exposure | Before CI surgery |
| P18 | F | 50 | R | 22 | CI24RE (CA) | CP900 Series | 16 | Progressive | After CI surgery |
| P19 | F | 62 | R | 22 | CI24RE (CA) | CP810 | 30 | Undeveloped ossicles | Before CI surgery |
| P20 | F | 69 | R | 22 | CI512 | CP900 Series | 25 | Unknown | Before CI surgery |

*Note.* CI = cochlear implant; M = male; F = female; R = right; L = left; CA = Contour Advance.
